# Autologous haematopoietic stem cell transplantation for refractory stiff-person syndrome: the UK experience

**DOI:** 10.1007/s00415-020-10054-8

**Published:** 2020-08-12

**Authors:** Lewis Kass-Iliyya, John A. Snowden, Alice Thorpe, Helen Jessop, Andrew D. Chantry, Ptolemaios G. Sarrigiannis, Marios Hadjivassiliou, Basil Sharrack

**Affiliations:** 1 Academic Department of Neurosciences, Sheffield Teaching Hospitals NHS Foundation Trust, Sheffield, UK; 2 Department of Haematology, Sheffield Teaching Hospitals NHS Foundation Trust, Sheffield, UK; 3 Department of Oncology & Metabolism, University of Sheffield, Sheffield, UK; 4 University of Sheffield, Sheffield, UK; 5 The Academic Unit of Medical Education, University of Sheffield, Sheffield, UK

**Keywords:** Stiff person syndrome, Stem cell transplantation

## Abstract

**Abstract:**

Stiff Person Syndrome (SPS) is a rare immune-mediated disabling neurological disorder characterised by muscle spasms and high GAD antibodies. There are only a few case reports of autologous haematopoietic stem cell transplantation (auto-HSCT) as a treatment for SPS.

**Objective:**

To describe the UK experience of treating refractory SPS with auto-HSCT.

**Methods:**

Between 2015 and 2019, 10 patients with SPS were referred to our institution for consideration of auto-HSCT. Eight patients were deemed suitable for autograft and four were treated. Of the treated patients, three had classical SPS and one had the progressive encephalomyelitis with rigidity and myoclonus variant. All patients were significantly disabled and had failed conventional immunosuppressive therapy. Patients were mobilised with Cyclophosphamide (Cy) 2 g/m^2^ + G-CSF and conditioned with Cy 200 mg/kg + ATG followed by auto-HSCT.

**Results:**

Despite their significantly reduced performance status, all patients tolerated the procedure with no unexpected toxicities. Following autograft, all patients improved symptomatically and stopped all forms of immunosuppressive therapies. Two patients were able to ambulate independently from being wheelchair dependent. One patient’s walking distance improved from 300 meters to 5 miles and one patient’s ambulation improved from being confined to a wheelchair to be able to walk with a frame. Two patients became seronegative for anti-GAD antibodies and normalised their neurophysiological abnormalities.

**Conclusions:**

Auto-HSCT is an intensive but well tolerated and effective treatment option for patients with SPS refractory to conventional immunotherapy. Further work is warranted to optimise patient selection and establish the efficacy, long-term safety, and cost-effectiveness of this treatment.

**Electronic supplementary material:**

The online version of this article (10.1007/s00415-020-10054-8) contains supplementary material, which is available to authorized users.

## Introduction

Stiff person syndrome (SPS) is a rare autoimmune neurological disorder characterised by progressive axial muscle stiffness, central nervous system hyper-excitability, and stimulus sensitive painful muscle spasms. Needle electromyography (EMG) often shows continuous motor unit activity at rest [1, 2]. The combination of these features represents the classical form of SPS which is associated with antibodies against glutamic acid decarboxylase (anti-GAD) in around 70% of cases [3].

Other variants include focal or segmental SPS (stiff limb or stiff trunk), para-neoplastic SPS and progressive encephalomyelitis with rigidity and myoclonus (PERM), which in addition to the classic symptoms of SPS, manifests with brainstem signs, hyperekplexia, myoclonus, ataxia and dysautonomia. PERM is associated with anti-glycine receptor antibodies and is reported to be more responsive to immunotherapy [4–6]. Stiff Person Spectrum Disorder has recently been suggested as an overarching term to encompass the various clinical presentations of this condition.

The direct pathological role of the anti-GAD and anti-glycine receptors antibodies is uncertain. The immune-mediated pathogenesis of SPS is evidenced by co-existing autoimmune diseases and partial response to treatments such as intravenous immunoglobulin (IVIG), plasmapheresis and other immunosuppressive therapies including rituximab, mycophenolate and azathioprine [4]. Symptomatic improvement can be achieved using agents such as diazepam, dantrolene, gabapentin or baclofen. Nonetheless, SPS remains a significantly disabling condition with over half of patients requiring long term mobility aids [7].

Autologous Haematopoietic Stem Cell Transplantation (auto-HSCT) has been reported as a treatment option in a limited number of SPS patients with promising results [8]. Here we describe the UK’s experience in using auto-HSCT to treat patients with refractory SPS.

## Methods

Between 2015 and 2019 ten patients with SPS were referred to our institution, one of three national referral centres in the UK, for consideration of auto-HSCT from different UK and European centres. Patients’ clinical characteristics and outcomes are summarised in Table 1. All patients were assessed in a joint neurology and haematology transplant clinic. Before considering auto-HSCT the following criteria needed to be met: (1) established diagnosis of SPS; (2) significant disability secondary to SPS; (3) failure of at least one form of immunotherapy; and (4) absence of significant co-morbidities that would increase mortality risk associated with auto-HSCT. Funding requests from the NHS were made for UK patients.

**Table 1:** Summary of patients’ demographics, clinical phenotypes, neurophysiological and serological profiles, treatments tried and outcomes of patients with Stiff Person Syndrome (SPS) referred for consideration of auto-HSCT

Patient	Age/gender	SPS phenotype	Co-morbidities	EMG/blink reflex	Antibodies	Immunotherapy tried	Disease duration before HSCT	Neurological outcome after HSCT
A	36/F	Classical SPS	None	Continuous motor unit activity/blink reflex hyperexcitability	GAD > 2000 U/ml	IVIG Plasmapheresis	8 years	Two years from HSCT: Marked clinical improvement (wheelchair to independent walking) No further immunotherapy needed Anti-GAD and EMG remain positive
B	48/F	Classical SPS	Pulmonary sarcoidosis Type 1 diabetes Peripheral neuropathy	Continuous motor unit activity/blink reflex hyperexcitability	GAD > 2000 U/ml	IVIG Rituximab	4 years	One year from HSCT: Marked clinical improvement (wheelchair to independent walking) No further immunotherapy needed Anti-GAD and EMG became negative
C	37/F	Classical SPS	Type 1 diabetes	Continuous motor unit activity/blink reflex hyperexcitability	GAD > 2000 U/ml	IVIG Rituximab	9 years	Nine months from HSCT: Marked clinical improvement (from walking 300 meters to 5 miles) No further immunotherapy needed Anti-GAD and EMG remain positive
D	52/M	PERM & Gluten ataxia	Pulmonary embolism	Blink reflex hyperexcitability	GAD 372 U/ml Glycine positive Anti-gliadin positive	IVIG Plasmapheresis	5 years	Three years from HSCT: Partial clinical improvement (wheelchair to frame) No further immunotherapy needed Anti-GAD, anti-glycine and anti-gliadin became negative Blink reflex normalised
E	44/M	Classical SPS	Type 1 diabetes Gluten sensitivity	Continuous motor unit activity/blink reflex hyperexcitability	GAD > 2000 U/ml	IVIG Plasmapheresis Mycophenolate	7 years	Not transplanted as condition stable on mycophenolate
F	70/F	Classical SPS	Type 1 diabetes Hypothyroidism Coeliac disease Bronchiectasis with haemophilus colonisation and lobectomy.	Continuous motor unit activity	GAD > 2000 U/ml	IVIG (not tolerated) Azathioprine Methotrexate	20 years	Not transplanted due to co-existing lung disease
G	47/M	PERM	Recurrent thrombosis of AV fistula.	Continuous motor unit activity in paraspinal muscles	GAD negative Glycine negative	IVIG Plasmapheresis Azathioprine Mycophenolate	9 years	Not transplanted Funding declined
H	53/F	Classical SPS	Type 1 diabetes Hypothyroidism Gluten sensitivity Psoriatic arthropathy Sacral abscess Recurrent sebaceous cysts	Continuous motor unit activity/blink reflex hyperexcitability	GAD > 2000 U/ml Anti-TPO 397 U/ml	IVIG	15 years	Not transplanted Funding declined Later died from pneumonia (autopsy was not done)
I	35/F	Classical SPS	Deep venous thrombosis Heparin-induced thrombocytopenia	Continuous motor unit activity	GAD > 2000 U/ml	IVIG Plasmapheresis	2 years	Not transplanted Ongoing assessment
J	48/F	Classical SPS	None	Continuous motor unit activity	GAD > 2000 U/ml	IVIG Mycophenolate	3 years	Not transplanted Ongoing assessment

*PERM* Progressive Encephalomyelitis, Rigidity and Myoclonus, *Auto-HSCT* autologous haematopoietic stem cell transplantation, *GAD* glutamic acid decarboxylase, *EMG* electromyography, *IVIG* intravenous Immunoglobulin

Patients deemed suitable for auto-HSCT underwent detailed assessments including MRI of the brain and spine, nerve conduction studies, needle EMG to assess spontaneous motor unit activity and blink reflex study to assess brainstem hyperexcitability. Autoimmune screening included antinuclear, para-neoplastic, anti-GAD and anti-glycine antibodies as well as immunoglobulins and protein electrophoresis. Gluten sensitivity screening was undertaken including anti-gliadin antibodies, anti-TTG antibodies and anti-endomysial antibodies. This is because there is an overlap between anti-GAD associated disease and gluten sensitivity [9]. Infection screening included HIV, Hepatitis B & C, VZV, CMV, EBV, Toxoplasmosis and VDRL. Other baseline pre-transplant assessments included echocardiogram and pulmonary function tests.

Of the 10 patients referred, one was found to be stable on mycophenolate and was declined transplant (patient E), and another was declined due to significant co-morbidities conferring an unacceptable risk (patient F). Eight patients were deemed suitable for auto-HSCT. Two patients did not proceed to transplant because funding requests were declined by their health authority (patient G and H). Patient H subsequently died from a chest infection. Two patients are currently being assessed (patient I and J).

Four patients proceeded to auto-HSCT (patient A, B, C and D). Patient A, B and C had classical SPS. Patient D had the PERM variant of SPS.

In accordance with current auto-HSCT guidelines [10] patients received a standard regimen, with stem cell mobilisation consisting of cyclophosphamide 2 g/m^2^ and G-CSF followed by apheresis to achieve a minimum CD34+ stem cell dose of 2 × 10^6^/kg. Auto-HSCT conditioning regimen was cyclophosphamide 200 mg/kg (total dose, given as 50 mg/kg over days − 5 to − 2) with rabbit anti-thymocyte globulin (ATG, Thymoglobulin) total dose 6.0 mg/kg (given over days − 5 to − 2 as 0.5, 1.0, 1.5 and 1.5 mg/kg respectively with methylprednisolone cover) after which autologous peripheral blood stem cells were infused (on day 0). This is a non-myeloablative regimen which is similar to the one used by Dr Burt in Chicago for the treatment of this condition except that rituximab was not included in our regimen [11].

Data related to the duration of hospital stay, engraftment periods and complications of those who proceeded to auto-HSCT are summarised in Table 2. All patients were followed every 6-9 months in a joint neurology and haematology clinic.

**Table 2:** Summary of data relating length of hospital stay, engraftment time and complications of autologous haematopoietic stem cell transplantation (auto-HSCT) in the four patients treated for refractory stiff person syndrome

Patient	Age/Gender	Complications during priming and harvesting	Engraftment time after auto-HSCT (neutrophils > 0.5 × 10^9^/L and platelets > 20 × 10^9^/L)	Length of hospital stay for auto-HSCT	Required blood products/ transfusions	Complications during auto-HSCT	Long term sequelae
A	36/F	Headache E.coli UTI Pain	Neutrophils: 13 days Platelets: 12 days	26 days	Yes	Enterococcus UTI Pulmonary embolism Mucositis and rectal bleeding Post-menopausal symptoms	Alive with no complications
B	48/F	None	Neutrophils: 11 days Platelets: never dropped below 50	18 days	No	ESBL UTI Transient exacerbation of diabetes due to steroids	Alive with no complications
C	37/F	Gram-negative pantoea agglomerans from Hickman line-treated successfully with antibiotics	Neutrophils: 14 days Platelets: 13 days	21 days	Yes	Febrile neutropenia covered with antibiotics Transiently deranged LFTs Transient CMV and EBV viraemia	Alive with no complications
D	52/M	None	Neutrophils: 11 days Platelets: 10 days	16 days	Yes	Coagulase-negative staphylococcus line infection Transient EBV viraemia URTI (RSV)	Alive with no complications

## Transplanted patients

### Patient A

36-year-old female with no past medical history developed lower limbs and para-spinal muscle spasms that progressed over 3 months. Severe muscle spasms leading to arching of her back were triggered by sudden noise or cutaneous touch. Her symptoms continued to progress and she became wheelchair-bound 6 months later.

MRI of the neuroaxis and CSF examination were normal. Anti-GAD antibodies were positive (> 2000 U/ml). She was diagnosed with the classical form of SPS. She responded partially to plasmapheresis at the referring centre but continued to require very frequent treatments and was therefore started on IVIG.

**Fig. 1:**
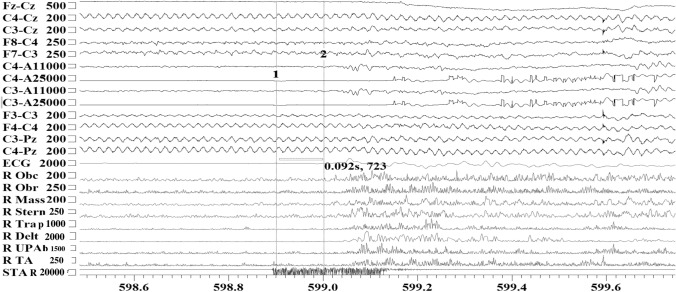
EEG/EMG polygraphy recording of patient A with classical stiff-person syndrome capturing exaggerated startle response to an unanticipated auditory stimulus. Low intensity unanticipated auditory stimulus around 50 dB elicited prominent muscle jerks (within 92 ms from stimulus presentation) followed by protracted spasms in multiple muscle groups. *Obc* orbicularis Oculi, *Obr* orbicularis oris, *Mass* massetter, *Stern* sternocleidomastoid, *Trap* trapezius, *Delt* deltoid, *UPA* upper abdominals, *TA* tibialis anterior, *STAR* sensor marking the onset of the unanticipated auditory stimulus

**Fig. 2:**
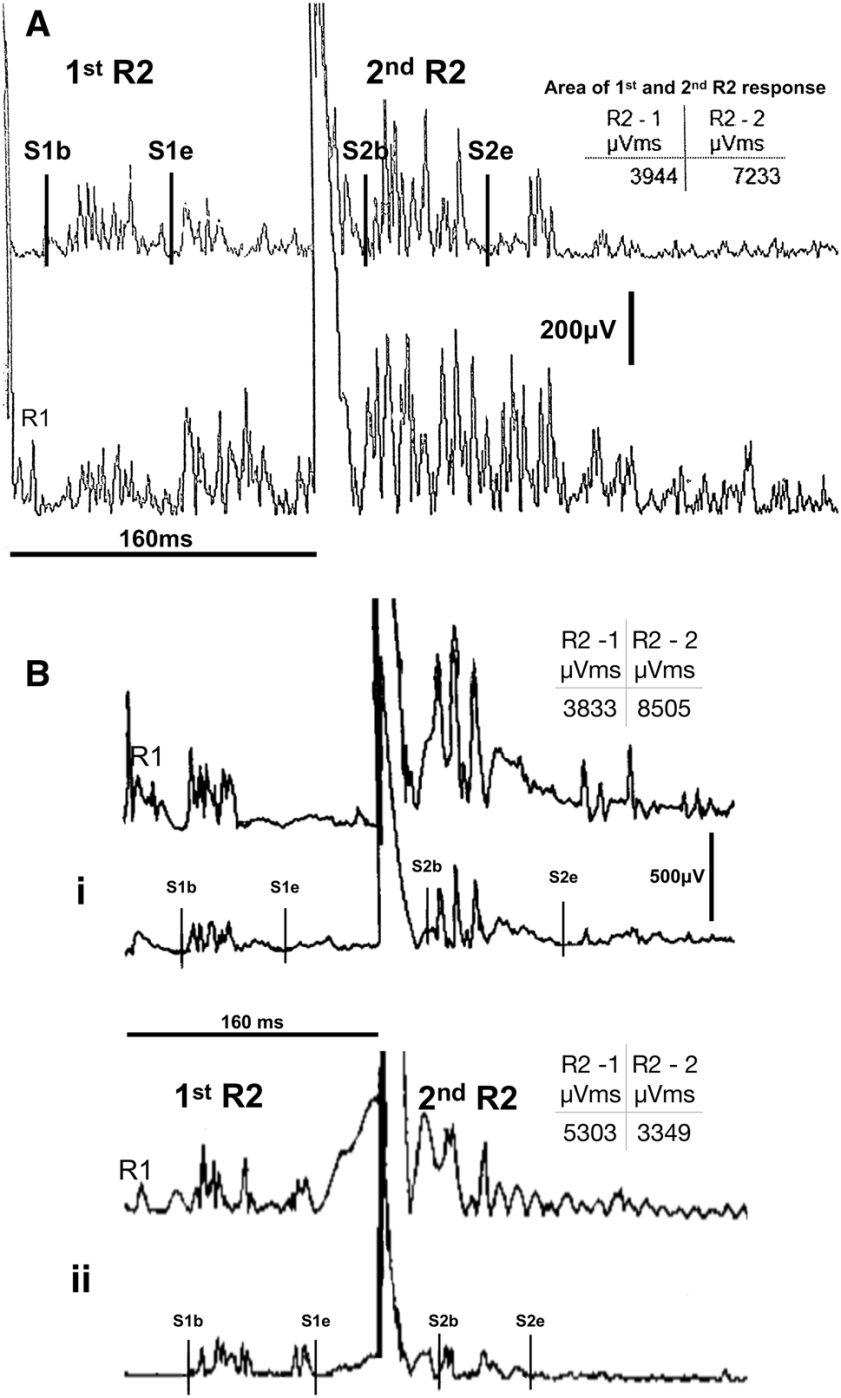
Blink reflex excitability studies at the short inter-stimulus interval between a conditioning and a test stimulus after 160 ms. Single square pulse electrical stimulation of the supraorbital nerve on one side is given at 20–25 mA and 0.2 s pulse width. The polysynaptic R2 response which is recorded following a test electrical stimulus from the contralateral side is typically supressed at such small interstimulus interval in healthy subjects. The least affected by artefact, rectified R2 waveform contralateral to the site of stimulation was used for analysis. In both patient A and patient B (classical stiff person syndrome) the contralateral R2 component that follows the test stimulus is enhanced (area estimates for R2 between cursors S1b/S1e and S2b/S2e are shown in the relevant embedded tables). The pre-HSCT study of patient A shows clear enhancement of the R2 response that follows the test stimulus in comparison to the earlier R2 waveform that followed the conditioning stimulus. For patient B, comparison between the pre-HSCT (Bi) and post-HSCT (Bii) examination shows relative normalisation of blink reflex excitability in the latter; the R2 area following the test stimulus is relatively suppressed in comparison to the R2 area of the conditioning stimulus (Bii). This electrophysiological assessment is used as a semiquantitative assessment of brainstem excitability

When she was reviewed at our institution, she was severely disabled by her symptoms requiring regular IVIG treatments at a dose of 90 g every 12 days. She was taking regular diazepam at a dose of 30 mg per day and morphine up to 60 mg a day to control pain. On examination, she had brisk reflexes and severe clonus. She was exquisitely touch-sensitive which induced severe prolonged painful muscle spasms. The muscle spasms were severe enough to compromise her breathing and she required intermittent oxygen. EMG showed continuous muscle fibre activity. Blink reflex study was abnormal with marked amplification of the R2 component recorded following test stimulus in keeping with brainstem hyperexcitability (Fig. 2A). Given her extreme stimulus sensitivity, she underwent EEG/EMG polygraphy recording which captured exaggerated startle response to auditory stimuli in keeping with brainstem hyperexcitability (Fig. 1). The rest of her work-up and immunology screen were negative.

She received auto-HSCT 8 years from the onset of her symptoms. Transplantation proceeded with no unexpected complications apart from routine toxicities (Table 2).

When reviewed 5 months after auto-HSCT, the majority of her neurological symptoms had improved significantly. She was ambulating independently and required no further IVIG but continued to take diazepam. She had mild startle responses. Anti-GAD antibodies remained positive at > 2000 U/ml.

Repeat EMG undertaken a year post-auto-HSCT remained abnormal with continuous motor unit activity. Blink reflex study with short interstimulus intervals was not possible as immediately following the first electrical stimulus protracted contraction of facial muscle tended to emerge. Therefore, no meaningful comparison could be made with the previous study. Overall, the neurophysiological assessment continued to show features in keeping with SPS despite the clinical improvement.

She was reviewed again 2 years following auto-HSCT and was noted to have remained off all immunotherapy and was able to walk independently. She reported occasional muscle spasms affecting her arms and legs and poor exercise tolerance. Neurological examination was normal. She declined repeat neurophysiological assessment. Her anti-GAD antibodies remained positive.

### Patient B

Forty-eight-year-old female with a past medical history of type 1 diabetes, diabetic neuropathy and pulmonary sarcoidosis presented with intermittent muscle spasms affecting all four limbs. The spasms progressed gradually causing increasing difficulties with her mobility over a period of 4 years. At that point, she was mostly wheelchair-bound and only able to ambulate indoors with the help of a frame (supplementary electronic material). Painful muscle spasms were triggered by cutaneous touching. Prior to her referral to our institution she had been diagnosed with stiff person syndrome and was started on IVIG which helped her symptoms. However, she was requiring an infusion every 2 weeks. Subsequently, two doses of rituximab were given which improved her symptoms but did not reduce the need for regular IVIG. She was reliant on diazepam and baclofen for symptomatic relief.

When reviewed at our institution she was noted to have intermittent sustained muscle spasms on examination. She also had clinical signs of length-dependent peripheral neuropathy which was confirmed on nerve conduction studies. EMG displayed continuous motor potential activity and blink reflex demonstrated brainstem hyper-excitability with lack of suppression of R2 component following the test stimulus (Fig. 2Bi). Anti-GAD antibodies were positive (> 2000 U/ml) and the rest of her immunology screen was negative. Infection screening prior to auto-HSCT identified hepatitis B core antigen positivity. In the absence of any risk factors, this was thought to be caused by repeated IVIG infusions. Hepatitis B DNA PCR was negative.

Auto-HSCT was undertaken at our institution 4 years into her illness and progressed uneventfully (Table 2). Hepatitis B DNA PCR was pre-emptively monitored throughout her immunosuppression and remained negative.

When reviewed in clinic 6 months after auto-HSCT, her muscle spasms were noted to have improved significantly. She required no further doses of IVIG but continued to use a small dose of Baclofen. She was no longer requiring a wheelchair and started walking with the support of a stick. She continued to suffer from fatigue.

Repeat EMG showed significant improvement with the patient being able to completely suppress all motor unit potential activity in muscles that were previously affected by severe stiffness. The blink reflex excitability studies also improved with a more suppressed R2 component following the test stimulus compared to the R2 component from the conditioning stimulus (Fig. 2Bii). She was able to walk 10 meters in 15.2 s with a stick.

Serologically, anti-GAD antibodies reduced from > 2000 to < 0.5 U/ml.

A year after auto-HSCT her marked improvement continued. She was no longer reporting any spasms and was able to walk independently for long distances (Electronic supplementary material). Her diabetes control also improved and she came off all her anti-diabetic treatments. She walked 10 meters in 9 s without assistance or stopping. A repeat EMG at that point showed no evidence of stiff person syndrome. Anti-GAD antibodies remained negative.

### Patient C

Thirty-seven-year-old female with a history of type 1 diabetes. She developed progressive painful muscle spasms affecting her core musculature and limbs, which were not controlled despite high doses of diazepam (30 mg/day) and gabapentin (2700 mg/day). Nine years into her symptoms she was able to walk unaided for a maximum of 300 meters. She struggled with social anxiety due to muscle spasms, which progressed to affect her face and jaw.

Prior to her referral for auto-HSCT the patient had received five courses of IVIG, which provided transient benefit. She had three infusions of rituximab, which did not relieve her symptoms. Azathioprine was not tolerated.

Neurological examination showed marked stiffness of her abdominal and para-spinal muscles. EMG showed continued motor activity in the para-spinal muscles. Blink reflex study showed evidence of brainstem hyperexcitability. Anti-GAD antibodies were positive (> 2000 U/ml). The rest of the immunology, paraneoplastic and infective screens were negative. Auto-HSCT was offered 9 years after symptom onset. There were no major complications from auto-HSCT apart from routine toxicities (Table 2).

Nine months after transplantation the patient reported marked improvement of the severity of her muscle spasms and stiffness. She reported mild fatigue but she was able to walk for up to 5 miles a day. She continued to use diazepam and gabapentin albeit at much lower doses (10 mg of Diazepam/day and 500 mg of Gabapentin/day). Blink reflex study did not show evidence of brainstem hyperexcitability however, limited lumbar paraspinals EMG (patient was needle phobic) showed continued motor potentials. Anti-GAD antibodies remained positive after transplantation.

### Patient D

52-year-old male with no significant past medical history presented with progressive asymmetrical muscle stiffness affecting initially the right leg but subsequently other parts of his body. The stiffness progressed over 5 years to involve all four limbs, which significantly impaired his ability to carry out the activities of daily living. Facial muscles involvement interfered with speech and swallowing and he occasionally bit his tongue.

**Fig. 3:**
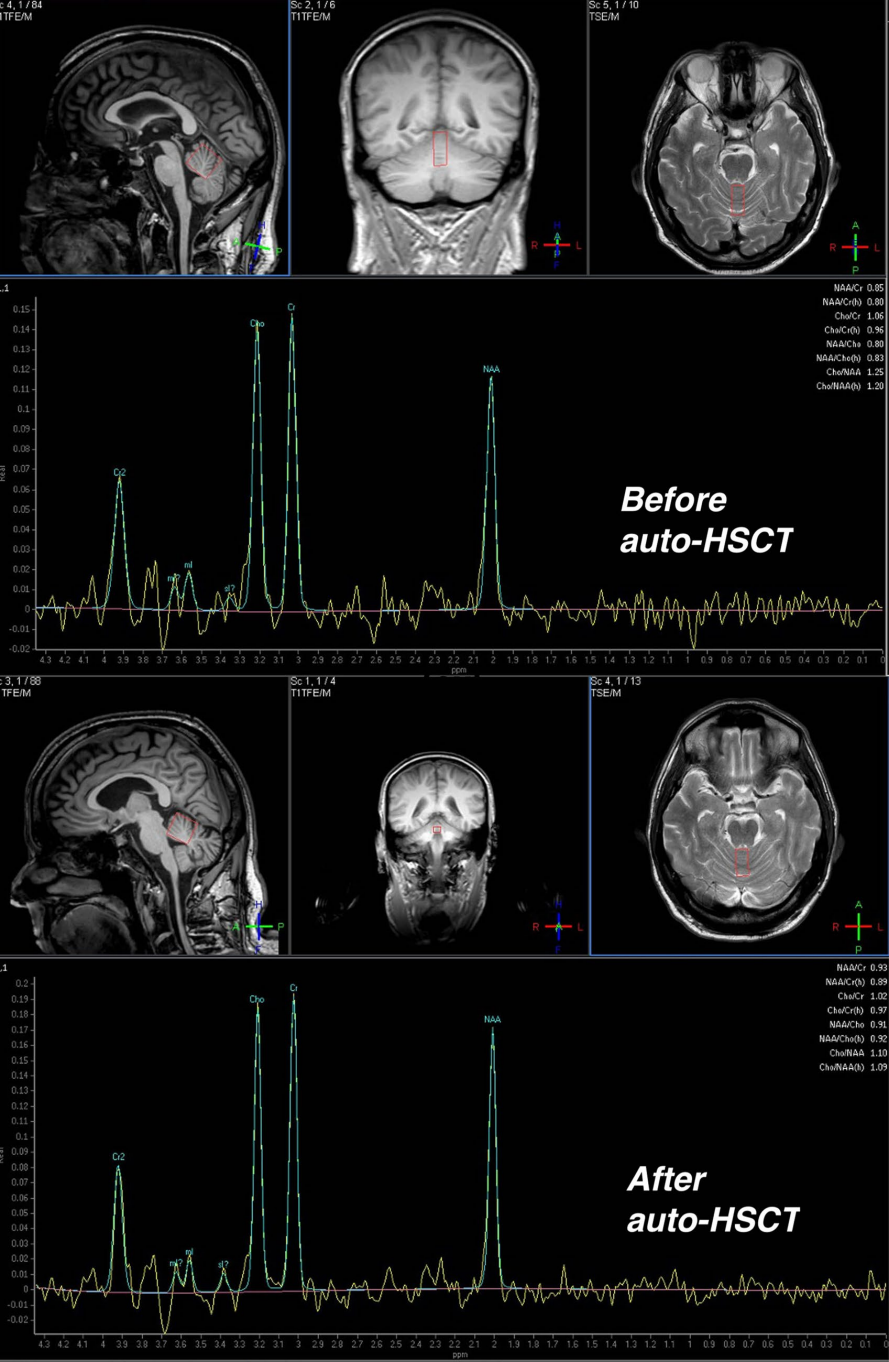
MRI spectroscopy of 52-year-old male with stiff person synonym (PERM - patient D) who underwent autologous haematopoietic stem cell transplantation (auto-HSCT). The MRI demonstrate cerebellar involvement showing NAA/creatine ratio of 0.85 from the superior vermis (normal above 1.00) before auto-HSCT which improved to 0.93 after auto-HSCT

Examination revealed marked muscle rigidity, brisk reflexes and clonus. MRI of the spine showed moderate spondylosis which did not account for the patient’s symptoms. Serological testing for gluten sensitivity revealed positive anti-gliadin antibodies and a gluten-free diet was adopted. MRI of the brain showed mild atrophy of the cerebellar hemispheres. MRI spectroscopy demonstrated low NAA/Creatine ratio of 0.85 from the superior vermis (normal over 1.00) (Fig. 3). CSF examination was normal.

Nerve conduction studies were normal. EMG did not show continuous motor unit activity however, blink reflex studies showed evidence of hyper-excitability. Anti-GAD antibodies were positive at 372 U/ml. Anti-glycine antibodies were positive. Paraneoplastic, anti-NMDA and anti-VGKC antibodies were negative.

He was diagnosed with the PERM variant of SPS on the basis of the clinical features and the serology results. He was started on IVIG which resulted in partial clinical improvement. However, he continued to require infusions at a dose of 150 g every 3 months. He could not tolerate Diazepam, Baclofen, Tizanidine or Dantrolene. He did not tolerate mycophenolate which he tried for 2 months. Over the subsequent years, he became wheelchair-bound and dependent on IVIG which he continued for a year.

Auto-HSCT was offered 5 years after symptom onset. While he was being considered for auto-HSCT he developed deep vein thrombosis and a large saddle pulmonary embolism thought to be related to poor mobility and regular IVIGs. He was started on rivaroxaban. Auto-HSCT proceeded uneventfully apart from routine toxicities (Table 2).

Four months after auto-HSCT, his mobility improved from wheelchair to a frame. His legs remained stiff but arms improved significantly so he was able to feed and wash himself. Anti-GAD and anti-glycine antibodies became negative. Repeat blink reflex study post-transplantation showed no evidence of hyperexcitability. He was no longer requiring regular IVIG. His speech remained dysarthric but his swallowing normalised. He was no longer biting his tongue. He stopped all regular medications.

Two years after auto-HSCT he remained off IVIG and had good use of his upper limbs but continued to use a walking frame. EMG and blink reflex studies remained normal and anti-GAD was negative. MRI spectroscopy of the cerebellum showed improvement of his NAA/creatine ratio (Fig. 3).

## Discussion

We report our experience in using auto-HSCT to treat four patients with refractory SPS.

All four patients experienced marked improvement in their symptoms and mobility following treatment. In spite of clinical improvement, patient A and C (classical SPS) continued to have high circulating anti-GAD antibody titre. EMG and blink reflex excitability assessment remained abnormal in patient A but normalised in patient C. On the other hand, patient B (classical SPS) and patient D (PERM) became seronegative for circulating antibodies and their EMG and blink reflex studies normalised. Furthermore, MRI spectroscopy values in patient D improved following treatment.

Significantly, with respect to both clinical impact and health resource utilisation all our patients stopped regular IVIG and other forms of immunotherapy with sustained symptomatic and clinical improvement.

The response to auto-HSCT confirms the autoimmune basis of SPS. Continued seropositivity for anti-GAD in half of our patients is comparable to the previous two case reports of using auto-HSCT to treat SPS [8].

The role of anti-GAD in the pathogenesis of SPS remains uncertain. GAD is the rate-limiting step in the decarboxylation of L-glutamate to γ-aminobutyric acid (GABA). Thus, anti-GAD antibodies are postulated to lead to decreased levels of GABA in the brainstem and spinal cord resulting in dis-inhibition and hyper-excitability [12]. However, several observations question anti-GAD pathogenicity in SPS. These include lack of correlation of antibody titres and disease severity [13], absence of anti-GAD antibodies in some SPS patients [14] and reports of clinical improvement with ongoing high circulating antibodies [8].

Interestingly patient B who became anti-GAD negative after auto-HSCT also reported improvement of her diabetic control. Thus, anti-GAD may support the diagnosis of SPS and other autoimmune dysfunction but does not fully explain the pathophysiology.

Glycine receptors are inhibitory receptors found on the neuronal cell surface predominantly in the brainstem and spinal cord. They exert their effects through chloride current resulting in membrane hyperpolarisation and reduction in excitation [15]. Antibodies against the alpha-1 subunit of glycine receptors are, therefore, associated with hyperexcitability. Null mutations in glycine receptors result in hereditary hyperekplexia characterised by an excessive startle and often muscle rigidity [16].

When anti-glycine receptor antibodies are present, they are typically associated with the PERM variant of SPS [5]. However, anti-glycine receptors antibodies are also found in around 15% of patient with classical SPS patients with uncertain significance [17]. Furthermore, glycine receptor antibodies have been reported to occur in other autoimmune conditions with heterogeneous phenotypes including ataxia, limbic encephalitis and myoclonic epilepsy [16].

The pathogenic roles of B cell and T cell immunity in SPS are similarly not well defined. Intrathecal production of oligoclonal anti-GAD IgG antibodies is continued by active B cells with the help of T cells that are activated by neural antigens [18]. Thus, immuno-ablative therapy to eliminate the dysfunctional immune response is expected to offer benefit. Immuno-ablative chemotherapy is followed by the re-introduction of autologous stem cell graft aiming to restart a new self-tolerant immune system. The CSF was not assayed for the presence of anti-GAD antibodies in our patients, but this should be considered in future studies to assess whether this parameter would correlate with treatment response.

At the time of writing of this case series all our transplanted patients manifested sustained clinical improvement without the need for any form of immunotherapy. Follow-up post-transplant has ranged from 12 months to 3 years. No patient encountered major or unexpected complications. Longer-term benefit of auto-HSCT in SPS remains to be ascertained.

Autologous HSCT has shown promise as a treatment option for a range of treatment-refractory autoimmune neurological conditions such as multiple sclerosis, neuromyelitis optica, myasthenia gravis and chronic inflammatory demyelinating polyneuropathy [10, 19]. Our experience further supports its use for refractory stiff-person syndrome. Auto-HSCT may prove to be a more cost-effective treatment in patients requiring regular treatment with expensive modalities, such as IVIG. Further work is warranted to establish long-term safety, efficacy and cost-effectiveness of auto-HSCT in SPS, along with optimising patient selection and transplant technique. This calls for collaboration between centres that provide this service.

## Electronic supplementary material

Below is the link to the electronic supplementary material.

Supplementary material 1 (MP4 82628 kb)

Supplementary material 2 (MP4 5421 kb)

## Data Availability

Available.
